# Endoscopic Sphincterotomy Before Cholecystectomy?

**DOI:** 10.1155/1989/63874

**Published:** 1989

**Authors:** Peter B. Cotton

**Affiliations:** Division of Gastroenterology Duke University Medical Center P. O. Box 3341 Durham North Carolina 27710 USA


### ENDOSCOPIC SPHINCTEROTOMY BEFORE CHOLECYSTECTOMY?

### ABSTRACT

*J.P. Neoptolemos, D.L. Carr-Locke and D.P. Fossard.* (1987) Prospective randomised study of preoperative endoscopic sphincterotomy versus surgery alone for common bile duct stones. British Medical Journal, 294, 470–474.

**One hundred and twenty patients with known common bile duct stones were entered into a prospective randomised study of preoperative endoscopic sphincterotomy and stone clearance (group 1) versus surgery alone (group 2). Five patients were incorrectly entered; the 55 patients randomised to group 1 and the 60 randomised to group 2 were well matched with respect to clinical features and biochemical and medical risk factors. In group 1 endoscopic stone clearance was successful in 50 patients (91%); five of these patients refused elective surgery, though this was subsequently necessary in one. In group 2 common bile duct stones were cleared surgically in 54 of 59 patients (91.5%); one patient was treated by endoscopic sphincterotomy alone because of a myocardial infarct. The overall major complication rate in group 1 was 16.4% and included two deaths; in group 2 this was 8.5% and included one death. The minor complication rate in group 1 was 16.4% and that in group 2 13.6%. These differences in outcome were not significant.**

**Despite a significant reduction in total hospital stay of patients in group 1, these results do not support the routine use of preoperative endoscopic sphincterotomy in patients having biliary surgery for stones in the common bile duct.**

### PAPER DISCUSSION

David Carr-Locke and his surgical colleagues from Leicester, England have made several major contributions to our understanding of the role of endoscopic techniques in the management of patients with biliary tract obstruction. In the early 1980's, whilst most other groups were just getting started or simply counting numbers, Carr-Locke set up studies to look carefully and prospectively at several of the major clinical questions including, for instance, the role of urgent endoscopic management in patients with acute cholangitis, and in acute pancreatitis related to gallstones[1,2]. At that time it was already clear that most retained stones could and should be treated endoscopically[3]. There were preliminary reports suggesting that duct stones should be dealt with by endoscopy even when the gallbladder had not been removed[4]. This was recommended for elderly and high risk patients in whom the duct stones were the primary cause of symptoms. The rationale was to defuse the clinical situation and proceed either to elective cholecystectomy, or a "wait and see approach". This approach has been vindicated by subsequent followup studies which show that cholecystectomy has been necessary in only 10–20% of such patients in periods of up to 10 years[5–7]. It was then a small and seductive step to suggest that *all* patients requiring surgery for gallstones with some suspicion of duct stones should undergo prior ERCP (with sphincterotomy if stones were found), leaving only a simple cholecystectomy for the surgeon. Bile duct exploration adds considerably to the morbidity of biliary tract surgery, and the literature can be construed to suggest that the combined morbidity and mortality of endoscopic sphincterotomy and cholecystectomy might be less than that of standard cholecystectomy with duct exploration[8]. Carr-Locke bravely set out to test this hypothesis in a randomized study with the ready collaboration of his surgical colleagues in the Leicester area. Over a period of 4½ years (from April 1981) they entered 120 patients with bile duct and gallbladder stones into a prospective study in which patients were allocated randomly to undergo either endoscopic stone extraction followed by surgery, or surgery alone. The two groups were well matched with respect to clinical features, biochemical and medical risk factors. As is clear from the published abstract, there were no major differences between the two groups in terms of successful duct clearance, complication rates, and deaths. The authors therefore conclude that their results do not support the routine use of preoperative endoscopic sphincterotomy in patients having biliary surgery for stones in the common bile duct and gallbladder.

This study was carefully performed, well analyzed, written and discussed. Is the result definitive, or will some endoscopists still feel justified in cleaning the common duct endoscopically in patients who are fit for orthodox surgery (and do not have acute cholangitis or pancreatitis)?

The main drawback to the study is that it's design almost guaranteed a negative result. Patients were excluded from the study if they were "unfit for surgery". These patients went directly to endoscopic treatment for their bile duct stones; cholecystectomy was not performed unless and until gallbladder complications developed. The number of patients so excluded is not stated, but may well have been more than the patients randomized, in view of the other publications from this group during the same period. This means that the study really tested only the ability of the clinicians involved to select out (for endoscopic treatment alone) those patients who would develop more complications at surgery. A more academic (if uncaring and probably unethical) protocol might have placed the threshold for surgical risk at a different level. This would have included more frail patients and probably produced a result more in favor of the endoscopic approach.

The authors add important caveats. It has been shown conclusively in other studies that endoscopic sphincterotomy followed by cholecystectomy is a logical approach to patients who have a temporary contraindication to surgery whether this is due to the illness caused by bile duct obstruction (cholangitis or pancreatitis)[1,2], or to some unrelated medical problem (such as pregnancy or recent myocardial infarction). Most endoscopic and surgical series do not define these factors adequately, so that there is great difficulty in comparing the results[8,9].

The study also highlights the problem of sepsis, and it is worrying that patients undergoing endoscopy had a significantly high incidence of pseudomonas cultures from gallbladder bile. This suggests that the disinfection procedures were not ideal.

A factor not considered by this group were the potential *long-term* risks of the two different approaches. Endoscopic sphincterotomy permanently damages the sphincter, which might have long-term consequences. A followup study of 148 patients who had undergone endoscopic sphincterotomy for retained common bile duct stones (postcholecystectomy) 5–10 years previously showed a recurrent biliary problem rate of 13% (a mixture of stenosis, stones and cholangitis without obstruction)[10]. Whilst none of these complications were serious, they have to be weighed in the balance. There are relatively few data on the long-term followup of patients who have undergone surgical exploration of the bile duct, but further problems are not unknown[11]. It should also be remembered that the improved drainage produced by endoscopic sphincterotomy might *reduce* the risk of further stone recurrence. I hope that the Leicester group will be following these patients and report again on their status at five to ten years.

The goal posts have been moved since this study was started – and are still moving. New treatments for gallbladder stones (specifically external lithotripsy and percutaneous dissolution) add new opportunities for confusion, discussion and study. Hopefully, groups such as Carr-Locke's will define appropriate questions and attempt to answer them in a scientific manner.

It may be helpful to conclude by categorizing different clinical scenarios. Patients with acute biliary obstruction due to stones should be treated initially by endoscopic means as a matter of urgency if obstruction persists – whatever their age and premorbid state of health. Patients with less acute symptoms but who are seriously unfit for surgery should also be treated endoscopically; the gallbladder can be dealt with as a separate issue later when necessary. Patients with bile duct and gallbladder stones who have no contraindication to surgery are well managed by an orthodox operative approach. Those patients who seek a nonoperative alternative will be considered for protocols of external lithotripsy or dissolution methods, along with endoscopic management of duct stones. The goal posts will move again when chemists develop agents capable of dissolving or fragmenting mixed stones. It will then be unnecessary to perform a sphincterotomy to clear the common duct, since placement of a double lumen nasobiliary tube will permit appropriate perfusions. Experiments are even being made to place catheters endoscopically through the cystic duct into the gallbladder for stone dissolution.

**Keywords:** Gallstones; sphincterotomy, ERCP.

Peter B. Cotton
Division of Gastroenterology
Duke University Medical Center
P. O. Box 3341
Durham, North Carolina 27710
U.S.A.

### REFERENCES

1. Neoptolemos, J.P., Carr-Locke, D.L., Leese, T. and James, D. (1987) Acute cholangitis in association with acute pancreatitis: incidence, clinical features and outcome in relation to ERCP and endoscopic sphincterotomy. *British Journal of Surgery,* **74**, 1103–6
2. Neoptolemos, J.P., Carr-Locke, D.L., London, N.J., Bailey, I.A., James, D., and Fossard, D.P. (1988) Controlled trial of urgent endoscopic retrograde cholangiopancreatography and endoscopic sphincterotomy versus conservative treatment for acute pancreatitis due to gallstones. *Lancet,* **2**, 979–83
3. Cotton, P.B., and Vallon, A.G. (1981) British experience with duodenoscopic sphincterotomy for treatment of bile duct stones. *British Journal of Surgery,* **68**, 373–5
4. Cotton, P.B. and Vallon, A.G. (1982) Duodenoscopic sphincterotomy for removal of bile duct stones in patients with gallbladders. *Surgery,* **91**, 628–30
5. Cotton, P.B. and Dineen, L. (1989) Endoscopic treatment for bile duct stones without cholecystectomy. Follow-up at three to ten years. *Gastrointest Endosc* (in press)
6. Davidson, B.R., Neoptolemos, J.P. and Carr-Locke, D.L. (1988) Endoscopic sphincterotomy for common bile duct calculi in patients with gallbladders in situ considered unfit for surgery. *Gut,* **29**, 114–20
7. Ikeda, S., Tenaka, M., Matsumoto, S., Yashimoto, H. and Itoh, H. (1988) Endoscopic sphincterotomy and long-term results in 408 patients with complete followup. *Endoscopy,* **20**, 13–17
8. Cotton, P.B. (1984) Endoscopic management of bile duct stones (apples and oranges). *Gut,* **25**, 587–97

9. Little, J.M. (1987) A prospective evaluation of computerized estimates of risk in the management of obstructive jaundice. *Surgery,* 473–6
10. Hawes, R.H., Cotton, P.B. and Vallon, A.G. Follow-up at six to eleven years after duodenoscopic sphincterotomy for stones in patients with prior cholecystectomy. *Gastroenterology* (in press)
11. Larson, R.E., Hodgin, J.R. and Priestly, J.T. (1966) The early and long-term results of 400 consecutive explorations of the common duct. *Surg Gyn Obst* **122**, 744–50

### CHOLECYSTOENTEROSTOMY OR CHOLEDOCHOENTEROSTOMY FOR DISTAL BILE DUCT OBSTRUCTION?

### ABSTRACT

*I. James Sarfeh, Eric, B. Rypins, James G. Jakowatz and George L. Juler.* (1988) A Prospective Randomised Clinical Investigation of Cholecystoenterostomy and choledochoenterostomy. The American Journal of Surgery, 155, 511–414.

**A prospective, randomized clinical trial was conducted to assess the efficacy of bilioenteric bypass in noncalculous distal biliary obstruction. Thirty-one patients required bypass for either malignant obstruction or chronic pancreatitis and were randomized into two groups: cholecystoenterostomy or choledochoenterostomy with cholecystectomy [15,16]. Nine bypasses failed after cholecystoenterostomy and two after choledochoenterostomy (p <0.04). Eight of the 9 failures occurred in the subgroup of 22 patients with malignant biliary obstruction. In this subgroup, five bypasses failed within 90 days of operation, all after cholecystoenterostomy (p = 0.03 compared with choledochoenterostomy). The results indicate that choledochoenterostomy is the superior operation for malignant distal biliary obstruction. Additional studies will be necessary to identify the procedure of choice for benign noncalculous obstructions.**

### PAPER DISCUSSION

The authors are to be congratulated for conceiving and carrying out a prospective, randomized investigation of the values and defects of cholecystoenterostomy and choledochoenterostomy as alternative bypass conduits for patients with obstructive jaundice. All too often, opinions are given about the value of a method of treatment which are not based on good statistical evidence. Futhermore, surgeons are commonly criticized for not using prospective, randomized trials.

Thirty-one patients have been included in their trial, 15 undergoing cholecystenterostomy (CCE) and 16 choledochoenterostomy (CDE). Twenty-two of the patients had malignant jaundice, the remaining nine jaundice secondary to chronic pancreatitis. In the malignant group, 19 patients suffered from pancreatic carcinoma. A variety of other procedures, including gastroenterostomy, pancreaticoduodenectomy, colonic resection and gastrectomy were performed as necessary on selected patients. Patients were randomized according to whether the last digit of their social security number was odd or even. To be included in the trial, the patient needed to have evidence of a dilated common bile duct, a gallbladder and

